# Characterisation of Particles Emitted during Laser Cutting of Various Metal Sheets and an Exposure Assessment for the Laser Operators

**DOI:** 10.3390/ijerph19169888

**Published:** 2022-08-11

**Authors:** Stine Eriksen Hammer, Johanne Østereng Halvorsen, Pål Graff, Torunn Kringlen Ervik

**Affiliations:** National Institute of Occupational Health, 0363 Oslo, Norway

**Keywords:** laser cutting, metal sheets, exposure levels, scanning electron microscopy, ICP-MS, X-ray diffraction, scanning mobility particle sizer

## Abstract

Laser cutting is used in many industrial settings to achieve precise cuts of metal sheets. Laser operators may be exposed to particles formed during cutting when opening the cabinet or when metal sheets are exchanged. To characterise the potential exposure, particles formed during laser cutting were studied with scanning electron microscopy equipped with an energy dispersive X-ray detector and an energy backscatter diffraction detector. The total concentration of particles (11–615 nm) was determined online with a scanning mobility particle sizer. The chemical composition of the particles formed during the cutting of the different metal sheets was determined by inductively coupled plasma mass spectrometry (ICP-MS). X-ray diffraction was applied to determine the phase composition. The occupational exposure was assessed gravimetrically and by ICP-MS for five laser operators handling different laser cutters, and materials and were found to be low. Agglomerates and aggregates of condensation particles were formed during laser cutting, independent of the sheet type. Iron, present as both magnetite and α-Fe, was the main element found in the particles formed when cutting steel sheets. The size of the particles generated was mainly below 300 nm. Open laser cutters may lead to higher metal exposures, which is especially relevant when cutting metal sheets containing heavy metals.

## 1. Introduction

Cutting metal sheets of various steel types, or nickel-(Ni), aluminium-(Al), and titanium-(Ti) sheets is widespread in different metal industries. Automated advanced cutting machines are used when manual cutting methods are unsuitable or ineffective. These advanced cutting machines include, but are not limited to, mechanical cutting by abrasive water jet cutting as well as thermal cutting by plasma or laser beam. Laser cutting technologies with pollution strategies are summarised by He et al. [1]. Compared to the other techniques, laser beam cutting is cost-effective and can cut any complex material with a thickness up to 10 cm with high productivity, and leaves relatively thin kerf, yielding excellent precision of the cut [2]. An assistant gas jet removes the molten or vapourised material from the cut zone and can additionally act as an energy source. An inert gas, such as argon, provides mechanical force to eject material from the cut zone, while a reactive gas, such as oxygen, can cause an exothermic reaction to accelerate the cutting process [3]. The assist gas is chosen dependent on the material and whether cutting speed or cut edge quality is more important [2,4]. 

Condensation particles formed during thermal processes have been well characterised by welding [5], laser additive manufacturing [6,7,8,9], and in metal fume from other industrial production processes [10,11]. Generally, these particles are ultrafine agglomerates/aggregates of condensates. The detailed characterisation of particles formed during laser cutting is reported from cutting plastic [12] and composite materials [13]. The particle concentration and size distribution of the particles have been studied in the workroom air in relation to the laser cutting of metal sheets, but the concentrations in the study were influenced by other sources, such as trucks driving past the sampling equipment [14]. In a study of laser cutting in carbon steel, galvanised steel, and stainless steel, the particle number concentration increased with decreasing sheet thickness, and the median particle diameter was observed to be around 2–2.5 µm [15]. Waste products produced during the laser cutting of steel sheets have been thoroughly investigated with scanning electron microscopy (SEM)-energy dispersive X-ray (EDX) analysis, SEM-energy backscatter diffraction (EBSD), transmission electron microscopy (TEM), and X-ray diffraction (XRD) to investigate the possibility of reusing these particles in additive manufacturing [16]. 

Occupational exposure studies have been conducted for metal additive manufacturing in air [9,17] and by biomonitoring the workers’ blood and urine [18,19]. In all these studies, the air-dust level was low, and well below respective occupational exposure limits (OELs). Additionally, low levels of total Cr and hexavalent Cr were determined in the workroom air. Still, the reports showed increasing trends in urine Cr over one working week and in blood over one working year for additive manufacturers, indicating relevant exposure to this metal [18]. 

In this study, the physicochemical properties of particles emitted during the cutting of different metal sheets have been studied in detail. This includes the size distribution, number concentration, morphology, chemical composition, and mineralogy of particles formed during the laser cutting of metal sheets. Additionally, the size distribution of particles in the workroom air was studied together with an exposure assessment of particulate matter (PM) in three different laser cutting facilities in Norway. The purpose of the study was to gain knowledge about the particles emitted during laser cutting in regular production settings, which is important for risk assessment for workers in this industry. Knowledge of the potential exposure in these settings during laser cutting operations is central, as there are many different materials being cut with this technique, but very little is known of the potential exposure to laser operators.

## 2. Materials and Methods

### 2.1. Stationary Sampling

**Figure 1:**
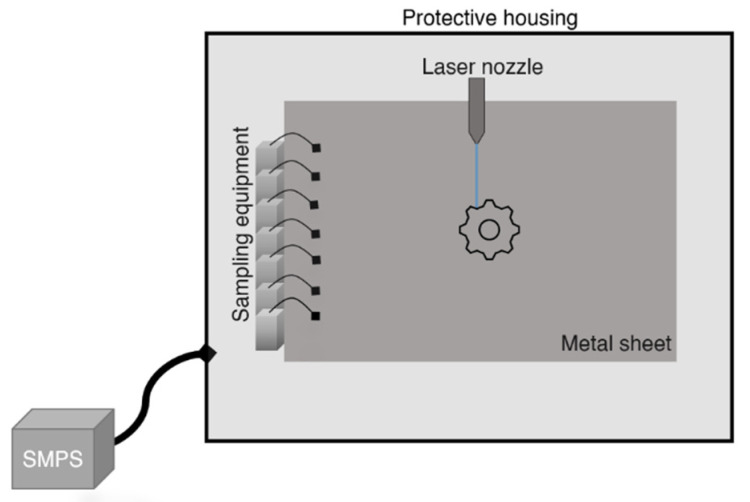
Scheme of the sampling equipment (total cassettes with filters and associated pumps) used during laser cutting. The antistatic tube connected to a scanning mobility particle sizer (SMPS) was attached to the inside of the protective housing.

Particle number concentration and size distribution were analysed in real-time with a TSI scanning mobility particle sizer (SMPS) instrument (model 3034, TSI Inc., Shoreview, MN, USA) in the electrical mobility diameter size range of 11–461 nm for the fibre laser, and 31–615 nm for the CO_2_ lasers, respectively. The particles were sampled from inside the protective housing of the laser cutter with the use of an approximately 2 m-long antistatic tube connected to the SMPS inlet. The sampling was performed during the cutting of 12 different metal sheets with four different laser cutters, one fibre laser (Bystronic, ByStar Fiber 6 kW), and three different CO_2_ lasers (Trumpf TruLaser 5030 classic, Bystronic Bystar 4020 and Bystronic Byflex), of which Bysonic Byflex is without protective housing. The fibre laser and Trumpf CO_2_ laser included a ventilation system, but the two other lasers had only one point of air extraction. The lasers were operating under normal drift ensuring exposure to relevant sampling. Particulate matter formed during the laser cutting was collected with the use of pumps built in-house (NIOH, Oslo, Norway) and SKC filter cassettes (SKC Inc., Eighty-Four, PA, USA) loaded with 25 mm polyvinyl chloride (PVC) membrane filters with a pore size of 5.0 µm (Merck Millipore, Burlington, MA, USA). This sampling equipment was placed inside the protective housing of the laser beam cutter (Figure 1). For Bysonic Byflex, the samples were collected as close as possible to the cut plates, approximately 0.5 m from the laser beam. In one of the workplaces, two lasers were operated at the same time. In this situation, the SMPS and APS were placed between the two CO_2_ lasers (Bystronic Bystar 4020 and Bystronic Byflex), approximately 1.5 m from each laser system. The sampling time was determined based on the cutting length of the same metal sheet type and included shifting sheets of the same type. The filter samples for gravimetric, elemental composition, and mineral phase determination of the bulk material were collected when the cutting time was longer than 30 min. Six parallel samples were collected for XRD analysis to provide the possibility of stacking filters on top of each other, and one sample was collected for ICP-MS analysis. This resulted in seven parallel samples for each metal sheet type. Additionally, to collect particles for microscopy, Cu transmission electron microscopy (TEM) grids with holey carbon films (EMresolution, Sheffield, UK) were affixed to the surface of 25 mm Merck Millipore PVC membrane filters in open-faced SKC filter cassettes. Three samples were collected for each material to minimise the risk of overloading the filters, with a variable sampling time between one minute to 11 min and 30 s.

Information on the sheet type and the laser applied during cutting on the different sheets is presented in Table 1. TEM grids were collected as described and particle size and number concentrations were measured for all material types if not stated otherwise. 

An aerodynamic particle sizer (APS) instrument model 3321 (TSI Inc., Shoreview, MN, USA) was placed within two metres of the laser cutter. This allowed us to measure the concentration and size distribution of particles with an aerodynamic diameter of 0.542–19.8 µm in the workroom air in the different facilities during the laser cutting operations.

### 2.2. Personal Sampling

Personal samples were collected in the breathing zone of five laser operators during their work shifts, which lasted between 265 and 483 min, respectively. The respirable cyclones (JS Holdings, Stevenage, UK) were loaded with 37 mm FPVC filters with a pore size of 5.0 µm (Merck Millipore, Burlington, MA, USA). Battery driven SG5200 pumps (GSA Messgerätebau, Ratingen, Germany) assured an air flow on 2.2 L/min. The workers operated the laser cutter during the whole working day. This included, but was not limited to, computer numerical control (CNC) work, overseeing the process and taking care of error messages that might appear, sometimes assisting in changing the metal plates, and maintenance such as changing laser lenses, mirrors, and nozzles on CO_2_-lasers. The workers handled several metal plates during the working day, related to what was planned for that specific day. Two workers wore sampling equipment at sites A and B, and one did so at site C (Table 1). All subjects gave their informed consent for inclusion before they participated in the study. The study protocol was approved by the Norwegian Agency for Shared Services in Education and Research.

### 2.3. Scanning Electron Microscopy

The particles collected onto the TEM grids were investigated with a Hitachi SU6600 field emission SEM (Hitachi High-Tech, Tokyo, Japan) equipped with a Bruker EDX detector (Bruker Nano GmbH, Berlin, Germany) and a NORDIF electron backscatter diffraction (EBSD) detector (NORDIF, Trondheim, Norway). The particle morphologies were observed and imaged in secondary electron (SE) imaging mode at a working distance of 10 mm and an accelerating voltage of 15 keV. The element composition of the agglomerates/aggregates was determined by SEM-EDX point and area analysis for a selected number of particles per sample. The crystalline phase was determined with the use of the EBSD detector for 50–100 particles per sample according to the method described in Ervik et al. [20].

### 2.4. Gravimetric Analysis

The mass of the PM was determined gravimetrically with a Sartorius micro model MC5 balance (Sartorius AG, Göttingen, Germany). The filters were conditioned for a minimum of two days in a room dedicated to low filter mass measurements (relative humidity 40 ± 2%, temperature 20 ± 1 °C), and discharged by a ^210^Po source prior to weighing. The limit of detection (LOD), calculated as three times the standard deviation of six field filter blanks, was 0.005 mg.

### 2.5. X-ray Diffraction

Three parallel PVC filters were removed from the monitor cassettes and stacked in the XRD sample holder on a Si crystal zero-diffraction plate. The samples were analysed in a PANalytical X’Pert^3^ powder diffractometer, equipped with a PANalytical Empyrean X-ray tube (Malvern Panalytical, Malvern, Great Britain). In-house reference materials of magnetite and hematite were also analysed. The resulting diffractograms were examined in HighScore Plus software (Malvern Panalytical, Malvern, UK) which determined the background. The search-and-match function of the software was used to identify mineral candidates in the ICSD database. Element information from SEM and ICP-MS was used in the restriction settings to limit the number of candidates. 

### 2.6. Inductively Coupled Plasma Mass Spectrometry

All PVC filters, including those with PM and field blanks, were transferred into separate vessels and added to 2 mL aqua regia (1:3, 65% HNO_3_, 37% HCl), 0.05 mL 40% HF (A.R. grade Sigma-Aldrich, Merck KGaA, Darmstadt, Germany), and 200 µL internal standard (2 µg/mL Rhodium). The samples were digested using a Milestone mls 1200 digestion module (Milestone, Fatebenefratelli, Italy) and diluted to 15 mL with MilliQ water.

The quantification of Al, Cr, Cu, Fe, Mn, Ni, Pb, and Zn was performed with an Agilent 8800 QQQ ICP-MS (Agilent Technologies, Santa Clara, CA, USA). Multi-element calibration solutions (Spectrapure Standards AS, Oslo, Norway) were prepared with acid-matched matrix solutions. Mild steel welding fume (MSWF-1) reference material (HSE’s Science and Research Centre, Derbyshire, UK) was used for quality control (Table 2).

### 2.7. Statistics

**Figure 2:**
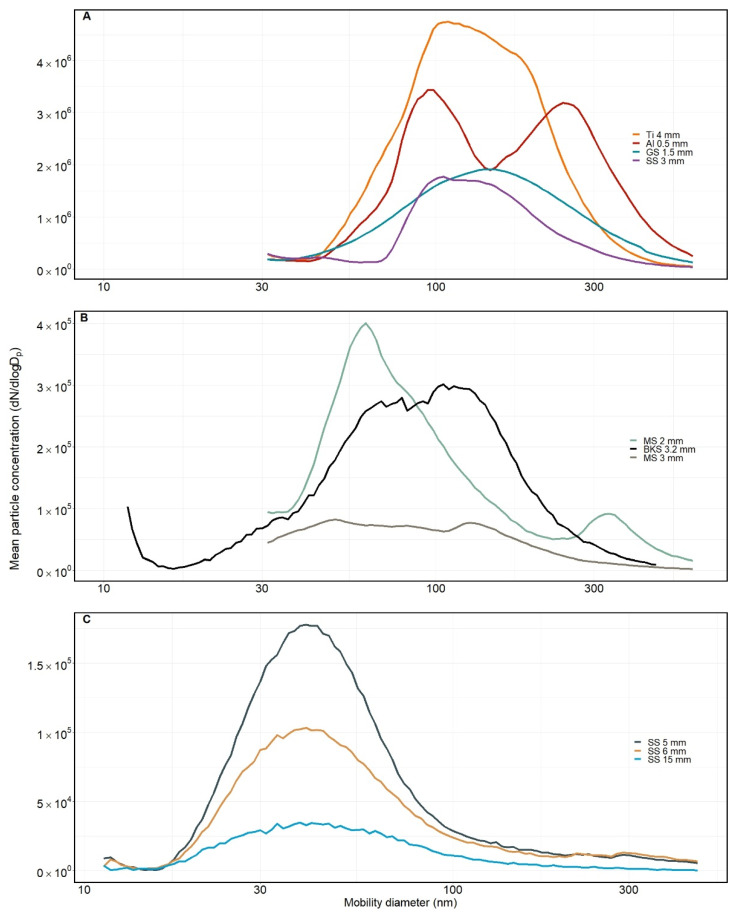
Particle size distribution for condensation particles from laser cutting of (**A**) aluminium- (Al), titanium- (Ti), galvanized steel (GS), and stainless steel (SS) sheets cut with a CO_2_ laser; (**B**) black steel (BKS) and mild steel (MS) sheets cut with fibre and CO_2_ laser, respectively, and (**C**) stainless steel (SS) sheets cut with a fibre laser.

Geometric mean, median, minimum, and maximum values are presented for PM dust level and the elemental concentration in the workers’ breathing zone because the variables are lognormal distributed. These are calculated using R studio version 4.1.2 (R core Team, Vienna, Austria) [21], and Figure 2 and Figure 5 are plotted with the additional CRAN packages ‘ggplot2′ [22],‘ggpubr’ [23], and ‘ggsci’ [24]. The limit of detections was calculated as three times the standard deviation of six blank samples. 

## 3. Results

### 3.1. Particle Number Concentration and Size Distribution

Mean particle concentration decreased with the increasing thickness of the metal sheet considering similar material types cut with a similar laser. This is illustrated for the SS sheets cut with a fibre laser (Figure 2C). When different material types and/or different lasers are considered, there are no obvious trends. The laser cutting of the 4 mm Ti sheet with a CO_2_ laser resulted in the highest mean particle concentration. Generally, the size distribution of the particles originating from cutting with the fibre laser of SS sheets was smaller than particles emitted during laser cutting with a CO_2_ laser. 

### 3.2. Particle Morphology

**Figure 3:**
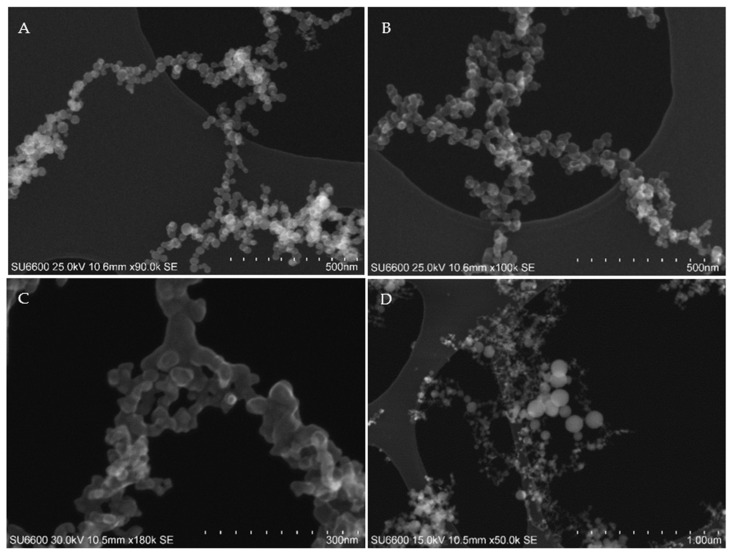
Secondary electron images of: (**A**) agglomerate of spherical primary particles, (**B**) agglomerate/aggregate of primary particles, (**C**) magnification of an aggregate, and (**D**) larger condensation particles.

The particles collected during the laser cutting of the various materials consisted of agglomerates of spherical primary particles (Figure 3A) and agglomerates/aggregate of coalescing primary particles (Figure 3B,C). A magnification of the aggregation is shown in Figure 3C. Most particles were in the size range of 5–50 nm; however, many of the samples also included some larger condensation particles with sizes ranging from 100–200 nm (Figure 3D). 

### 3.3. Chemical Composition of Condensation Particles

The amount (mg/m^3^) of PM was highest for the sample collected during the laser cutting of GS sheets (Table 3). As for the particle number concentration, the PM mass seems to decrease with increasing sheet thickness. One exemption is the sample collected during the CO_2_ laser cutting of the 3 mm MS which yielded the lowest mass concentration. 

**Figure 4:**
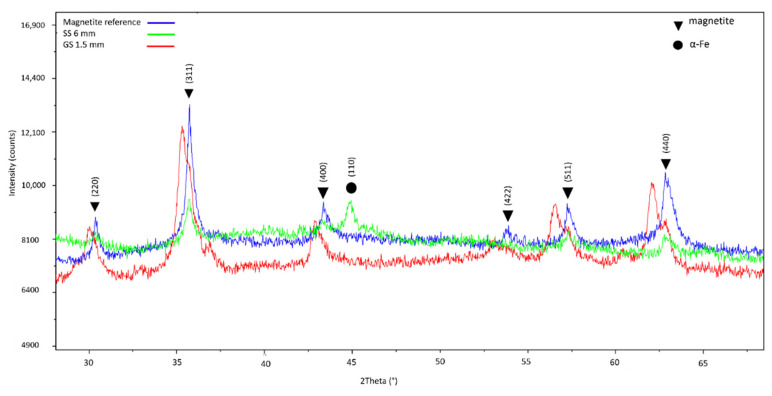
X-ray diffraction patterns for magnetite reference material and PM collected from cutting on GS 1.5 mm and SS 6 mm.

The XRD pattern for GS 1.5 mm, SS 6 mm and a magnetite in-house reference material, is shown in Figure 4. A good match was found between magnetite and the samples in terms of peak position and the relative intensity of the diffraction peaks. From the XRD pattern, it is shown that magnetite was found in both GS (red line) and SS (green line) samples, whereas α-Fe is only seen in the SS 6 mm sample. As indicated in Table 3, the GS sample was the only sample analysed with XRD where α-Fe was not found.

The XRD pattern for GS 1.5 mm, SS 6 mm, and magnetite reference material is shown in Figure 4.

### 3.4. Exposure Measurements

The exposure measurements indicate that the general exposure levels during laser cutting are low and below the current Norwegian OELs for all components, respectively (Table 4). The variation between workers can be explained by the fact that the samples were collected in different factories, each one cutting metal sheets for their own specific application. The open laser cutting of Pb containing BS pipes/sheets was performed by one of the workers, which resulted in a much higher air-Pb concentration measured in the breathing zone compared to the other workers.

**Figure 5:**
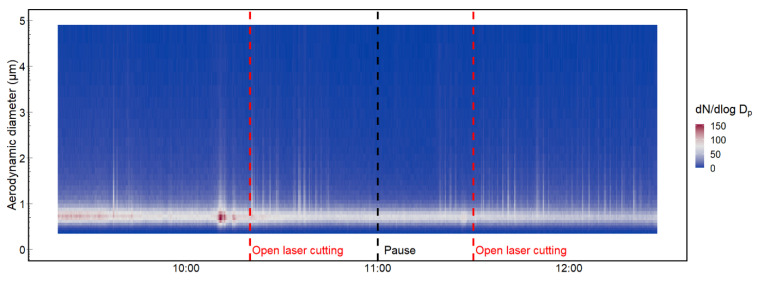
Particle size and number concentrations measured with APS during laser cutting. The red line indicates the start of cutting with the open laser and the black line indicates the stop. The closed laser cutter was operating in the background throughout the entire time.

The particle number concentration in the work environment during laser cutting with two laser cutters operating at the same time, one with and one without a cabinet, is presented in Figure 5. The laser cutter with a cabinet was cutting 10 mm Al sheets continuously between 09:20 and 12:27. The laser cutter without a cabinet was operated manually at intervals, for two minutes around 09:30, from 10:20 to 11:00 and from 11:30 to 12:30 (start-time marked in red, Figure 5). The steel tubes and sheets were manually handled between the laser cutting. The peaks of the higher concentrations of particles larger than 1 µm d_ae_ occurred frequently related to the laser cutting. However, the highest concentration of particles was measured before the manually handled laser cutting started, shortly after 10:00. During this time, the laser operator collected and prepared metal sheets for laser cutting. The higher concentration of particles, including particles larger than 1 µm d_ae_, indicates the resuspension of settled dust.

The size distribution of particles with a d_ae_ between 0.549–19.8 µm was similar in the workroom air of the three different factories, with the highest concentration around d_ae_ 0.9 µm [25].

## 4. Discussion

The particle number concentration of the condensation particles emitted during the cutting of metal sheets seems to be dependent on the thickness of the sheets when considering similar material types and similar laser types and/or laser beam energy (Figure 2). This result is consistent with what was reported by Pena et al. [15]. They explained this finding by suggesting that the excess energy from the laser cutting results in vaporisation rather than fusion. More energy is concentrated in less material in a thinner sheet, which leads to the high generation of aerosol. A similar trend is seen in the PM mass concentration (Table 3). However, it is not only the sheet thickness that influences the generation of particles. The heat-affected zone will also be influenced by other factors such as the composition of the metal sheets, cutting speed, and laser power [2]. Slower cutting may lead to more absorbed energy, when considering the same material, and may increase the width of the cut. On the other hand, cutting at high speed might result in an insufficient melting of bottom regions during the cutting and increase the risk of spatter from melted material. The speed and power must thus be adjusted according to the physical properties of the material, such as thermal conductivity and reflectivity. These properties affect the cutting process and thereby the generation of particles. A material with low thermal conductivity can be cut at a higher speed than materials with high thermal conductivity since the energy is concentrated in the cutting zone instead of being dissipated into the material [3]. In the present study, laser cutting into a 4 mm Ti sheet resulted in the highest number of condensation particles. This may be a result of the low thermal conductivity of Ti, which, as described in the thickness study by Pena et al. [15], will allow more energy to be concentrated in less material. In contrast, Al has a very high thermal conductivity and is reflective. However, the Al sheet was only 0.5 mm, which may be the reason why cutting this sheet resulted in the second highest particle number concentration. In general, the particle number concentration depends on the material properties, sheet thickness, laser power, cutting speed, and assistant gas.

Processes affecting particle size distribution are particle growth, agglomeration, source, and removal processes [8,26]. The smallest particle size distribution was observed for condensation particles from fibre laser cutting into 5 mm, 6 mm, and 15 mm thick SS sheets, yielding a maximum d_mob_ peak around of 40 nm. The size distribution of the particles collected during laser cutting with a CO_2_ laser in 3 mm SS sheets is higher, with the maximum peak around a d_mob_ of 200 nm. This may indicate that the laser type influences the size distribution of the condensation particles. The fibre laser is known to achieve a higher cutting speed compared to the CO_2_ laser, but a study by [27] illustrated that this only applied to sheet thicknesses below 6 mm when they tested SS material up to a thickness of 10 mm. Additionally, condensation particles from the fibre laser cutting of 3.2 mm BKS resulted in a larger particle size distribution than for the 5 mm, 6 mm, and 15 mm SS. This indicates that other parameters, like chemical composition, also influence the particle size distribution.

The collected particles show typical morphologies associated with thermal-generated condensation particles. The agglomerates/aggerates were often a mixture of agglomerates of loosely bound spherical primary particles (Figure 3A) and/or sintered or coalesce primary particles forming aggregates (Figure 3B,C), which is similar to the findings in Noskov et al. [7]. The size and shape of particles will depend on the relative rates of particle collision and coalescence. When coalescence is fast, spherical particles are formed, whereas if the collision rate dominates and coalescence is slow, fractal-like structures are formed [28]. These mechanisms will be determined by temperature and residence time. For laser cutting on steel sheets, the size of the primary particles may also be influenced by Fe vapour concentration. It has been suggested, by modelling fume formation in arc welding, that higher Fe concentrations result in larger primary particles while lower initial Fe concentrations result in smaller primary particles of Fe [29]. This also has consequences for the crystalline phases of particles as explained further below.

With SEM-EBSD, it was found that magnetite dominated the mineral phase for particles collected during the laser cutting of steel sheets. Magnetite was confirmed with XRD on bulk filter samples from SS 5 mm, SS 6 mm, BKS 3.2 mm, and GS 1.5 mm. Additionally, α-Fe was determined in the SS and BKS samples. Metallic Fe has earlier been observed in the core of condensation particles collected during additive manufacturing [7]. When particles are exposed to air at room temperature, an oxide shell will instantly be formed [30]. According to the model by Sanibondi et al. [29], nanoparticles in arc welding are formed mainly by FeO nucleation in the vapour phase, which oxidizes further to Fe_3_O_4_. Larger primary particles are formed when the Fe concentrations are higher. The larger particles may not be fully oxidized resulting in a core of α-Fe. Because SEM-EBSD was performed on relatively few selected particles, and because the EBSD signal only comes from the shell, α-Fe was not detected by EBSD. A crystalline pattern indexed as Hercynite, which is a spinel mineral with formula FeAl_2_O_4_, was detected with SEM-EBSD in the sample from cutting of 10 mm Al [31]. Both results from SEM-EDS and ICP-MS show that the PM also contained Cu. Substitution with Cu ions has earlier been found in this spinel phase [32]. Unfortunately, the Hercynite phase could not be confirmed with XRD as the diffractogram was indistinct. The amount of PM on the filter influenced the quality of the XRD diffractogram. TiO_2_ and Ti_2_O_3_ were indexed with SEM-EBSD in the sample collected from the Ti sheet cutting, but this was not confirmed with XRD as the cutting time was too short to collect enough material.

In general, the chemical composition of the condensation particles reflected the originating metal sheet, but there are some indications of contamination from previously cut sheets, from condensation particles formed during cutting with another simultaneous cutting laser or PM mixed in from the ventilation. An example of a sintered particle originating from a previously cut metal sheet is shown [25]. Another example of contamination is the relatively high Pb concentration measured in PM originating from 10 mm Al sheets (Table 3). These samples were collected at the same time as PTFE- and Pb-coated BKS pipe/sheets were manually handled on the laser without protective housing and cut only a few metres away. Another possibility may be that the samples were contaminated by fume and/or sputter/sintered particles from the grid supporting the metal sheet, or the workroom air entering via the ventilation system of the laser cutter.

A higher Mn/Cr ratio than in the originating metal sheet was found in the metal fume from laser cutting in SS (Table 2). Originally, the relative amount of Cr was about 18–20% in SS sheets and only 2% Mn. In the fume, on the other hand, there was about five times more Mn than Cr. This may be due to Mn being more volatile than Cr and readily forming oxides [8,33]. The enrichment of Mn in agglomerated ultrafine condensation particles was also reported by Ljunggren et al. [18], but the same was not reported for samples collected in the breathing zone of the laser additive manufacture workers in their study. In our study, however, a higher concentration of Mn than Cr was also found in the workers’ breathing zone. This may imply that the laser additive manufacturing workers were mainly exposed to the original powder, but the laser cutting workers in this study were exposed to the condensate particles formed during laser cutting.

The exposure levels to metals during laser cutting were below the respective OELs for the workers in the three different manufacturing plants. This is an unsurprising result, as the laser cutting was mostly enclosed in a cabinet and included point air extraction above the laser cutter or a built-in ventilation system. Further, concentrations of PM, Cr, Fe, Mn, Ni, and Pb were all well below what is reported in laser additive manufacturing [18]. Still, the particles generated during laser cutting are small, respirable particles which may penetrate deep into the lungs [34,35]. The exposure during open laser cutting was measured for one of the workers. This worker handled BKS tubes/sheets coated with PTFE and Pb. Laser cutting into this material resulted in a potential exposure to Pb of 0.01 mg/m^3^ which is below the current OEL of inorganic lead in workroom air. However, prolonged Pb exposure at these levels has been shown to be a risk for lead poisoning [36]. Measures should thus be taken to reduce the Pb exposure when laser cutting these materials. One limitation is the relatively scarce number of workers included in this study. Further research should be performed to get a better understanding of the exposure to laser operators, with a special focus on exposure during open laser cutting. Further studies should also include biomonitoring when handling material containing heavy metals such as Pb.

## 5. Conclusions

In this study, we found that the number of particles formed during laser cutting depended on the material type, thickness of the metal sheets, and the laser cutter characteristics. The size distribution may be dependent on the type of laser or the chemical composition of originating metal sheets. The laser cutting of the different sheets resulted in ultrafine agglomerates/aggregates of condensation particles, with the enrichment of more volatile compounds like Mn. The assessment of the workroom air collected in the breathing zone of laser cutting workers showed that the potential exposure to metals was low. However, the exposure to Pb-containing material during open laser cutting may potentially result in significant Pb exposure. Traditional occupational PM air measurements are mass-based, which may not be sufficient in this work environment, as most particles are in the ultrafine range. Future studies in this environment should include size to account for the exposure of particles contributing with very little mass. Additionally, biomonitoring of open laser operators may give a better indication of the extent of exposure.

## Figures and Tables

**Table 1:** Metal sheet information and laser cutting characteristics.

Material				Cutting Characteristics		Collection
Type	Thickness (mm)	Sheet Type	Additional Information	Laser Type	Time (min)	Site
Aluminium (Al)	0.5	5754 H34		CO_2_	4	B
Aluminium ^a^ (Al)	10	EN AW 1050A		CO_2_	187	C
Black steel ^a^ (BKS)	2.3	GGB DU	PTFE and lead coating	CO_2_	100	C
Black steel (BKS)	3.2	Hardox 450	Hot rolled steel	Fibre	54	A
Galvanized steel (GS)	1.5	DX51D+Z275		CO_2_	229	B
Mild steel (MS)	2	DC01AM	Cold-rolled steel covered with carbon-based oil	CO_2_	12	B
Mild steel (MS)	3	S355MC		CO_2_	78	B
Stainless steel (SS)	3	AiSi 316		CO_2_	9	B
Stainless steel (SS)	5	AiSi 304		Fibre	91	A
Stainless steel (SS)	6	AiSi 304		Fibre	200	A
Stainless steel (SS)	15	AiSi 316		Fibre	42	A
Titanium (Ti)	4			CO_2_	4	B

^a^ SMPS was not applicable due to restrictions from the factory.

**Table 2:** Recovery (%) of mild steel welding fume reference material.

Element	Recovery (%)
MSWF-1 Mild steel welding fume (*n* = 3)	
Fe	99 ± 5.3
Mn	101 ± 5.7
Zn	101 ± 4.6

**Table 3:** Geometric mean concentration (mg/m^3^) of particulate matter from seven parallel samples with minimum and maximum in brackets. Concentrations (µg/m^3^) of elements in one of these samples of particulate matter freshly emitted during the cutting of various sheets: aluminium (Al), black steel (BKS), galvanized steel (GS), stainless steel (SS), and mild steel (MS). The main mineral phase was determined by SEM-EBSD, and/or XRD, for three to four samples.

	GS 1.5 mm	MS 3 mm	BKS 3.2 mm	SS 5 mm	SS 6 mm	Al 10 mm	SS15 mm
Particulate matter ^a^	1.5 [1.2, 1.9]	0.1 [0.09, 0.13]	1.0 [0.7, 1.9]	0.8 [0.5, 1.2]	0.4 * [0.4, 0.5]	0.3 [0.2, 0.3]	0.4 ** [0.3, 0.4]
Al	2.4	2.1	5.5	5.7	5.8	40	4.2
Cr	0.3	0.04	1.9	1.2	0.5	0.3	0.5
Cu	1.2	0.05	4.3	0.6	0.3	20	0.4
Fe ^a^	0.7	0.05	0.3	0.4	0.2	0.04	0.1
Mn	3.3	1.1	9.9	5.6	2.6	1.0	2.1
Ni	0.2	0.2	1.7	0.7	0.4	0.2	0.5
Pb	0.008	0.002	0.1	0.03	0.02	11	0.03
Zn	<LOD	<LOD	<LOD	<LOD	<LOD	4.2	<LOD
Mineral phase	Magnetite ^b,c^	Magnetite ^c^	Magnetite ^b,c^ and α-Fe ^b^	Magnetite ^b,c^ and α-Fe ^b^	Magnetite ^b,c^ and α-Fe ^b^	Hercynite ^c^	Magnetite ^c^

^a^ mg/m3, ^b^ XRD, ^c^ SEM-EBSD, * n = 5, ** n = 6.

**Table 4:** Particulate matter and elemental concentrations [µg/m^3^] measured in the breathing zone of workers operating laser cutters, *n* = 5, and the current Norwegian occupational exposure limit (eight-hour).

	Median	Minimum	Maximum	Norwegian OEL (2022)
Particulate matter	52	29	115	5000 ^a^
Fe	5	2	14	3000 ^b^
Al	2	1	2	5000 ^c^
Zn	1	0.3	4	5000 ^d^
Ti	0.1	0.01	2	5000 ^e^
Mn	0.08	0.01	0.3	50 ^a^
Cr	0.07	<LOD	0.08	500 ^b^
Cu	0.03	0.02	24	100 ^c^
Pb	0.01	0.002	12	50 ^c^
Sn	0.002	<LOD	4	2000 ^b^

^a^ respirable dust, ^b^ not particle size dependent, ^c^ welding fume/smoke, ^d^ respirable as oxide, ^e^ as dioxide.

## Data Availability

The data presented in this study are available on request from the corresponding author.
